# Progress in Dermatology

**Published:** 1895-03-09

**Authors:** 


### PROGRESS IN DERMATOLOGY.

(*Continued from page* 369.)

**Alopecia.**—Hutchinson[47] describes two cases of alopecia, occurring at the early ages of two and three years respectively. There was no history of ringworm in either case, and both cases had existed many years. Hutchinson considers that the disease was contracted by direct contagion from a patient who had the same disease. Robinson[48] refers to the occurrence, on the scalp, of circumscribed bald patches, varying in size from a split pea to that of a shilling; the skin is completely atrophied and the hair never returns. He does not consider that the condition is due to a parasite, or is allied to alopecia areata, but, since the disease only occurs in women, he suggests that the lesion may be due to the result of direct injury to the hair bulbs caused by the use of curling irons, &c.

**Acne.**—Hodara[49] finds the following micro-organisms in acne pustules: (1) A form of coccus, (2) the large flask-shaped bacillus of Unna, and (3) a small bacillus; the third variety is, in his opinion, the probable cause of the disease. The flask-shaped bacillus is also found in pityriasis capitis, and in seborrhœic eczema. Dyer[50] recommends the internal treatment of acne by a pill of sulphide of calcium, or by ichthyol in five to ten drop doses. Stimulating local applications are given at the same time. Schaffer[51] describes an epidemic of miliaria in an Austrian village. The eruption consisted of numerous distinct red papules which itched considerably; they faded within a few hours, but left the patients weak for some time. Those who had recently suffered from boils escaped this epidemic. Crocker,[52] in a clinical lecture on diseases of the skin due to pus cocci, enumerates the following: Impetigo capitis, pustular eczema, sycosis, boils and carbuncles. The mode of inoculation, in the first named instances, is probably by means of the patient's nails. Van Hoorn[53] treats furunculosis in the following way: After aseptisising the boils and surrounding skin with a (1 in 1,000) solution of mercuric chloride, he covers the boil with a mercurial and phenol plaster. Lowenberg[54] uses a galvano-cautery, with a fine platinum point, which is introduced into the centre of the boil. This treatment is also useful for rebellious acne. Liddell[55] records two cases of sycosis which occurred on the arms and legs as well as on the face. He remarks on the rarity of such occurrence.

**Lymphangioma.**—Galloway[56] describes a case of this disease which occurred in a girl aged ten years. The eruption involved the skin over the central and upper parts of the left scapula, and consisted of numerous vesicles, containing clear fluid, arranged in groups. It had first been noticed at the age of three months, when the child had a fall; there was no vascular dilatation. The whole of the affected area was removed and a microscopic examination made. Renault[57] records a similar case, and recommends complete destruction of the growth with the actual cautery. Jamieson[58] also reviews another case, published by Elliott; the patient was thirty-nine years of age, and the vesicles appeared at the edges of the scars left by an attack of suppurative adenitis when the patient was a child.

**Thyroid Feeding.**—Abraham[59] reported three cases of lupus which had derived marked benefit from thyroid feeding; he also referred to two cases of leprosy treated by this drug. Cantrell[60] tried the remedy in four cases of psoriasis, and considers that if patients with psoriasis can take a sufficient dose they will be benefited. He found that a delicate youth could take a larger dose than a robust man. Crary[61] reviews the published cases of psoriasis treated with thyroid extract; he records nine cases under his own care, in only two of which was there any improvement. He does not think that there is any reason to believe that thyroid feeding is in any way curative of any real disease of the skin. Bettman[62] reviews all the cases of thyroid feeding which have been reported in the *British Medical Journal*.

**Therapeutics.**—Stewer[63] recommends thioform in a 10 per cent. ointment for the treatment of favus; the same ointment is useful in cases of moist eczema of the scalp or trunk. The action of this drug is more marked than that of iodoform. Leistikow[64] considers coal tar superior to all the tars. He has overcome the drawbacks to its use by using the following preparation: ℞. Ol. lith-anthracis, three parts; spiritus, two parts; ether sulph., one part. This is painted on the skin and dries very quickly. Sumergh[65] uses a soap containing naphthol, which possesses the advantage over coal-tar ointment of not acting as an irritant. He has employed it for pityriasis versicolor, herpes tonsurans, mycotic eczema, ephelis, acne, &c. The foam produced by the soap is allowed to remain on, for from five to twenty minutes; after its removal the affected skin is covered with powdered starch.

[47] Clin. Journ., Nov. 28th, 1894, p. 72. [48] Lancet, Sep. 8th, 1894, p. 573; [49] Am. Med. Surg. Bul., Oct. 15th, 1894. [50] Ther. Gaz., Oct. 15th, 1894, p. 691. [51] Am. Med. Journ., Oct., 1894, p. 478. [52] Clin. Journ., Dec. 19th, 1894, p. 127. [53] Brit. Med. Journ., ep., Nov. 17th, 1894, p. 79. [54] Ditto, ditto. [55] Ed. Med. Journ., Oct., 1894, p. 309. [56] Lancet, Nov. 23rd, 1894, p. 1,211. [57] Med. Week, Nov. 23rd, 1894, p. 573. [58] Ed. Med. Journ., Dec., 1894, p. 562. [59] Lancet, Dec. 22nd, 1894, p. 1,486. [60] Ther. Gaz., Sep. 13th, 1894, p. 600. [61] Med. Rec., Oct. 6th, 1894, p. 427, [62] Fortsch de Med., Oct., 1894, p. 743. [63] Med. Week, Oct. 16th, 1894, p. 527. [64] Brit. Med. Journ.. ep., Dec. 1st, 1894, p. 88. [65] Am. Journ. of Med. Sciences, Aug. 9th, 1894.

